# Breakdown of the Hospital System
*Correspondence is invited.


**Published:** 1889-03-09

**Authors:** B. Burford Rawlings


### Breakdown of the Hospital System.*

By B. Burford Rawlings.

(*Continued from page* 341.)

It will be not a little surprising to certain readers that a pamphlet bearing this ominous title should emanate from a provincial city, and record the failure of provincial philanthropy. We in London have been so long exposed to the influences of our own hospital question, and have become so habituated to its pertinacious familiarities, that we have learnt to look upon it as our particular inheritance—a metropolitan malady, interesting because unique, and respectable because colossal. Yet there is nothing in the Glasgow failure but a new illustration of an old truth. Like causes, generating under like conditions, necessarily produce like effects. In cities as large as Glasgow local sentiment droops with the roses, and, if it manage to live at all, it ceases to blossom. While the institutions of Little Pedlington occupy the main road, and are in the eye and mind of its inhabitants all the day long, those of a big town stand mostly in byeways or round corners, and neither fill a place in the thoughts of the population as a whole, nor secure the sympathies even of a neighbourhood.

After a perusal of this pamphlet, the authorities of metropolitan hospitals need no longer suppose they stand alone either in their anxieties or their shortcomings, and may take what comfort they can from this circumstance. Indeed an examination of Dr. Sutherland's figures at page 23 of his paper shows that while Glasgow, Liverpool, Sheffield, Manchester, Birmingham, and Edinburgh possess an aggregate population of 3,177,000, the accommodation of their voluntary hospitals is reckoned at 4,540 beds, and their hospital income at £198,313. The corresponding figures for the metropolis are a population of 4,500,000, 10,000 beds, and an income exceeding £700,000. The only town in which the hospital accounts do not show a deficit is Sheffield, and there the accommodation reckoned in beds is 1 in 830 of the population, as compared with 1 in 420 in London, and 1 in 312 in Edinburgh.

We note that in like manner with London, though necessarily in less degree, Glasgow suffers from the existence of small institutions—those stunted organisms which are chiefly lungs and stomach—and the most pregnant of the several interesting tables of figures which the author puts before us is the one which tells us that of 201 hospitals existing in the eleven largest cities of the kingdom, 105 contain less than 50 beds. Yet, with this plethora of "institutions" it is undoubtedly true that "no scientific attempt has been made to gauge the hospital wants of a community . . . . and, in consequence, in the great centres of population, the provision made for the sick poor is miserably inadequate." More than this, it is a remarkable fact that, although petty hospitals for a multitude of specialities abound, there is a terrible lack of accommodation for the victims of certain grave maladies more or less unfitted for the wards of general hospitals. How can it be otherwise, when a vast imperial work is undertaken piecemeal by a number of separate and impecunious organisations, among whom there is no collective action whatever? The first step towards making an adequate and scientific provision for the sick poor in London appears to be the suppression or absorption of redundant institutions, and the curtailment of individual liberty to foist new hospitals upon the community. This contention is by no means inconsistent with a conviction that the 10,000 beds contained in the voluntary hospitals are insufficient for the wants of the population, plus the many country patients who flock to London hospitals in cases of serious illness, and who necessarily occupy a far larger proportion of beds than their numbers considered alone would explain. We do not reckon the beds in the parish hospitals, because in the first place, as Dr. Sutherland points out, admission to them constitutes the patients paupers; and, in the second place, as he does not point out, the absence of a visiting staff, and the incompleteness of the medical and surgical arrangements render these hospitals, with rare exceptions, no better than the ordinary infirmaries of workhouses. These exceptions, if we accept the statement of Dr. Sutherland, must include the Union infirmaries of Liverpool and Birmingham. Unlikely as it appears, it is not impossible that the infirmaries of these towns receive a larger proportion of the non-pauper population than their voluntary hospitals, but to adduce as proof of this the larger number of deaths which occur in the former appears a method of reasoning as gruesome as it is fallacious. The voluntary hospitals will scarcely admit the soundness of the argument, while the workhouse infirmaries, which necessarily receive a larger number of aged persons, may well deprecate the manner of demonstrating their superiority.

Whatever the exceptions here and there, the undoubted fact remains that only the voluntary hospitals command the services of the most eminent members of the profession, and are stored with the capacity for dealing with cases of grave malady. The recollection of this accentuates the startling assertion that "in Scotland there are seventeen counties with an aggregate population of 725,000 persons that have not a single hospital bed." Picture the suffering—the remediable suffering to which the population is condemned by such a state of things. Could apathy much further go, or could the opponents of the voluntary system desire a deadlier weapon than is supplied in this statement? Well may Dr. Sutherland plead that, if the looked-for Royal Commission issue, the scope of its inquiry should not be limited to the metropolis.

The plan of admitting patients at the recommendation of subscribers is subjected to the adverse criticism it is sure to encounter when considered in the abstract. There is nothing to be said in its favour so far as it puts a difficulty in the way of the sufferer, but a great deal when by its means other and richer people, who seek to introduce patients and are apt to overlook obligations, are brought to book. Whatever their technical rights, we believe the subscribers would be among the first to recognise the impracticability and the barbarity of limiting the work of the hospitals to their nominees—in other words, of condemning to suffering or death those who are not provided with a subscriber's letter; and, illogical as the custom is, we doubt if any hospital of repute would reject an urgent case not so provided. Nevertheless, it is likely that the rule requiring a letter operates as a hindrance to that prompt application which in many instances is of vital importance.

It is impossible, within ordinary limits of space, to do more than touch thus lightly upon a few of the many points raised in this instructive pamphlet. Taking a bird's-eye view of the contents, we may say that, realising as we do the cogency of many of the author's arguments, we yet hesitate to adopt his conclusions. Moreover, when he discourses upon purely financial questions, we seem to detect deficiencies not previously apparent, and to miss the actual knowledge, personal experience, and familiarity with detail which have been conspicuous in his earlier pages. He shares the general objection to supporting our hospitals out of the rates, and echoes the prophecy so often before heard that as soon as rates are levied in aid, that moment philanthropy will come to an end; but he is sanguine than an Imperial subsidy would not have the same result. No reference is made to the practice so unequally and unrighteously enforced in London of rating hospitals to the poor, and we may therefore hope that the Glasgow authorities are innocent of that crowning iniquity. Neither do we find anything in the paper before us to indicate

* Correspondence is invited.

that the author appreciates the nature of the disheartening struggle in which every unendowed hospital is engaged in order to obtain even an insufficient income. Pages might be written in illustration of the waste of money and energy called for in this perpetual battle to keep open the doors. Does Dr. Sutherland believe these desperate efforts could be maintained concurrently with the receipt of a Government grant?

We have no experience of the methods of Dublin hospitals, and must therefore assume that the one illustration the kingdom affords of a joint support is strictly appropriate, but the common sense view would appear to be that a subsidy could not be accepted without a surrender of independence, and that must certainly mean, among other things, the crippling of powers now expended upon financial work. We are far from asserting that the loss of absolute independence would be in itself an evil; on the contrary, the establishment of the central authority which is so ardently desired by most students of the hospital question, would necessarily produce some curtailment of the liberty of individual organisations. The truth is that the subject is beset with difficulties, and it is not those who know most of hospital finance who pretend to see a clear way out of them, but rather those who are not restrained by any superabundant knowledge or encumbered with a sense of responsibility.

The wisest course at the present stage of hospital affairs seems to be to demand inquiry rather than to formulate specific remedies. Judged by reason alone, it seems indefensible that the performance of a great public duty should be left to private enterprise, with all its ascertained deficiencies. Yet there is a general consensus of opinion that it will be a misfortune if the philanthropic character of our hospitals be destroyed. In approaching the inquiry, which we are told impends, the hospitals need not lose heart. Inquiry will not only expose defects, but may be trusted to make manifest—possibly to the awakening of the public conscience—the good work performed and the ability to compass still greater ends when the conditions are more favourable. If knowledge is power, the hospitals are very far from powerless. Let them remain conscious of their strength and of their history. Equipped as they are with the only medical schools the country possesses, they are able to give back full value for whatever they receive. Is there no way of utilising the knowledge and the resources of our hospitals to their own support and improvement?

